# Mechanically Interlocked Molecules: A Supramolecular Computational Picture of Rotaxane‐Based Switches, Shuttles, and Tristable Devices in Solution

**DOI:** 10.1002/open.70291

**Published:** 2026-08-25

**Authors:** Costantino Zazza

**Affiliations:** ^1^ Department for Innovation in Biological Agro‐food and Forest Systems (DIBAF) Università della Tuscia Viterbo Italy

**Keywords:** DFT, mechanically interlocked molecules, molecular dynamics, noncovalent interactions, QTAIM

## Abstract

This contribution highlights a coherent series of recent computational studies devoted to the theoretical characterization of mechanically interlocked molecules (MIMs)—specifically [2]rotaxane molecular shuttles and switches—in organic solvents. Combining density functional theory (DFT) and all‐atoms molecular dynamics (MD) with the quantum theory of atoms in molecules (QTAIM) and the independent gradient model based on Hirshfeld partition (IGMH), a first‐principles picture of the noncovalent and covalent interactions, repertoire that governs macrocycle translocation, metal‐induced locking, and stimuli‐responsive switching, has emerged. Distinct rotaxane platforms in different exercise conditions suggest that theoretical investigations can quantitatively reproduce and rationalize conformational shaping and spectroscopic observables, thereby providing a design roadmap for next‐generation soft molecular machines of increasing complexity and functionalities. In this respect, the simulation scenario is further enriched by a self‐consistent graphical visualization of both covalent and noncovalent interactions combining new computational strategies like the interaction region indicator (IRI) and the local electron energy density *H*(***r***) (GLED) descriptors over supramolecular assemblies of hundreds of atoms at the DFT cost.

## Graphical abstract

This contribution highlights recent studies focused on mechanically interlocked molecules. By combining density functional theory‐converged wavefunctions with highly effective real‐space algorithms for visualizing and quantifying covalent bonds and noncovalent interactions, a first‐principles description of the driving forces governing molecular motion, locking, and switching may be obtained. The advanced descriptors herein reported enable a rational and predictive design of complex supramolecular machines.

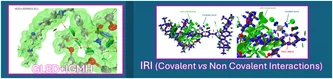

## 1 Introduction and Research Context

The dream of constructing machines that operate at the molecular scale—engines, switches, shuttles, and ratchets whose working strokes are individual conformational changes rather than the collective motion of macroscopic parts—has captivated chemists and physicists for decades [1, 2, 3]. It was elevated from speculation to experimental reality through the ingenious synthetic strategies of Jean‐Pierre Sauvage, J. Fraser Stoddart, and Ben Feringa, whose Nobel Prize in Chemistry in 2016 consecrated the field of molecular machines and mechanically interlocked molecules (MIMs) as one of the defining frontiers of contemporary science [4, 5, 6]. Among MIMs, rotaxanes, dumbbell‐shaped axle components threaded through one or more macrocyclic rings, with bulky stopper groups preventing dethreading have emerged as the canonical scaffold for molecular switches, shuttles, and motors [7, 8, 9, 10, 11, 12, 13, 14, 15, 16, 17, 18, 19, 20]. Their defining topological feature, the mechanical bond [1, 2, 3, 16], endows them with properties unreachable in conventional covalently bonded assemblies: the macrocycle can translate quasiunidirectionally along the axle, pirouette around it, or be locked at specific recognition stations, all without ever forming or breaking a single covalent bond between the ring and the thread.

From an applications standpoint, the payoffs of mastering rotaxane‐based motion at the nanoscale are substantial. Molecular shuttles in which the macrocycle position is switchable by pH or redox stimuli can act as mechanically gated carriers for drug delivery, as logic gates in molecular electronics, or as force‐generating elements in stimuli‐responsive materials and actuators [21, 22, 23, 24]. Tristable rotaxanes [25, 26, 27] that cycle through three discrete states under orthogonal external stimuli represent a step change in information density and functional complexity relative to bistable two‐state nanoscale size switches. Metal‐actuated rotaxanes open access to reversible, coordination‐chemistry‐driven control of mechanical motion, with potential applications in catalysis and sensing [28, 29, 30]. Realizing these promises requires, however, a degree of design precision that goes far beyond the trial‐and‐error of early rotaxane synthesis: one must be able to predict and finely tune the energetics and kinetics of macrocycle translocation, the selectivity of recognition‐site affinity, and the response of the system to environmental variables such as solvent polarity and temperature [31]. This is precisely where theoretical and computational chemistry becomes useful. The last two decades have seen a rapid evolution of the computational schemes available to study large supramolecular assemblies in solutions. Density functional theory (DFT) [32], particularly when augmented with empirical dispersion corrections (DFT‐D3) [33] and polarizable continuum solvation models (C‐PCM) [34], is now capable of delivering accurate geometries and interaction energies for systems containing several hundred atoms. Time‐dependent DFT (TD‐DFT) [35] connects electronic structure to spectroscopic observables, bridging computations and experiments through simulated UV–vis spectroscopic signatures. Classical all‐atoms molecular dynamics (MD), parameterized with modern force fields (OPLS‐AA) [36, 37, 38], captures the thermal fluctuations and entropic contributions that are invisible to static quantum‐chemical calculations. Enhanced sampling techniques such as umbrella sampling allow the extraction of free‐energy landscapes and activation barriers directly comparable to NMR kinetic data [39, 40]. Crowning this hierarchy, the quantum theory of atoms in molecules (QTAIM) [41, 42, 43] of Bader offers a rigorous, topological partition of the electron density, enabling a chemically transparent classification of every interaction—from strong covalent bonds through medium‐strength hydrogen bonds to weak van der Waals contacts—based on universal descriptors such as the electron density *ρ*(***r***), its Laplacian ∇^2^ *ρ*(***r***), and the local electronic energy density *H*(***r***). Complementary real‐space visualization tools, notably the noncovalent interaction (NCI) analysis [44] and its Hirshfeld‐partitioned variant IGMH [45, 46], translate these topological quantities into color‐coded three‐dimensional isosurfaces that map the full noncovalent interaction network in complex supramolecular aggregates.^1^

**SCHEME 1:**
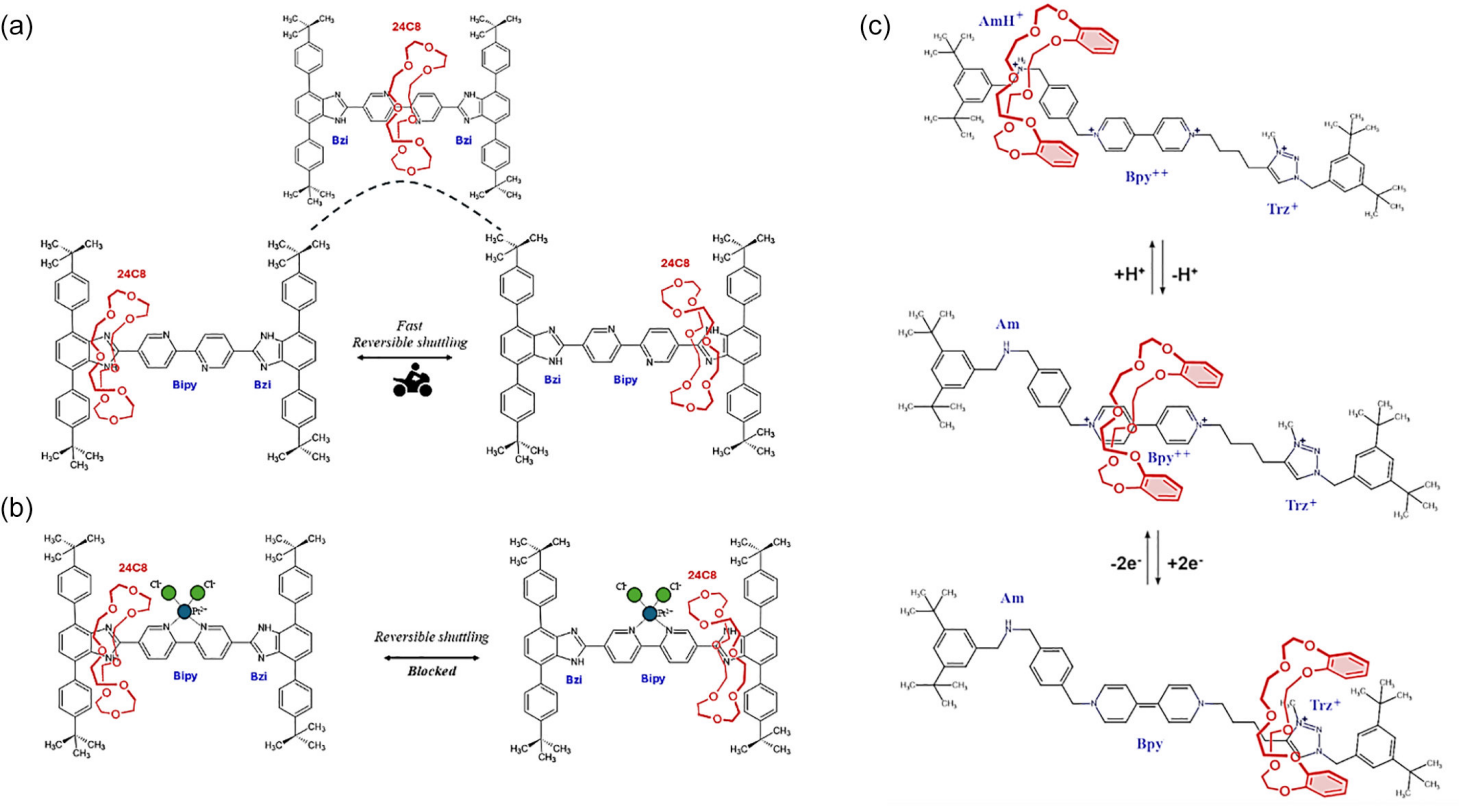
(a) Schematic view of the symmetric H‐shaped Stop‐[Bzi(24C8)‐Bipy‐Bzi]‐Stop [2]rotaxane working in CH_2_Cl_2_ at room temperature (shuttling rate of *ca*. 61.2 s^−1^). (b) The Stop‐[Bzi(24C8)‐Bipy(PtCl_2_)‐Bzi]–Stop complex having the effect of blocking the reversible shuttling in (a) [47, 48]. (c) The tristable [2]rotaxane as proposed by A. Credi and coworkers in Ref. [27]: the ammonium (Am^+^) and bipyridinium (Bpy^2+^) stations being responsive to orthogonal stimuli—pH variation and selective electrochemical reduction—allow a direct control in CH_2_Cl_2_ over the macrocycle (DB24C8) translocation along the functionalized “three stations” molecular thread.

The recent studies discussed here (see Scheme 1) illustrate the power of a multiscale, multimethod framework for unraveling the molecular mechanisms underlying function across distinct rotaxane architectures in organic solvents. Collectively they span: (i) QTAIM characterization of a rigid H‐shaped [2]rotaxane shuttle with a central 2,2′‐bipyridyl (Bipy) recognition/chelation unit, from both the crystallographic reference structure and a Zn(II)‐induced octahedral intermediate [47, 48]; (ii) all‐atoms MD free‐energy profiling and DFT + QTAIM analysis of the same system extended to the PtCl_2_‐locked, translationally inactive form^2^ [49]; and (iii) a full DFT/TD‐DFT/QTAIM + IGMH investigation of a recently synthesized pH‐ and redox‐driven tristable [2]rotaxane incorporating three orthogonally addressable recognition stations [27, 50].

## 2 QTAIM Analysis of an H‐Shaped [2]Rotaxane with a 2,2′‐Bipyridyl Core

**FIGURE 1:**
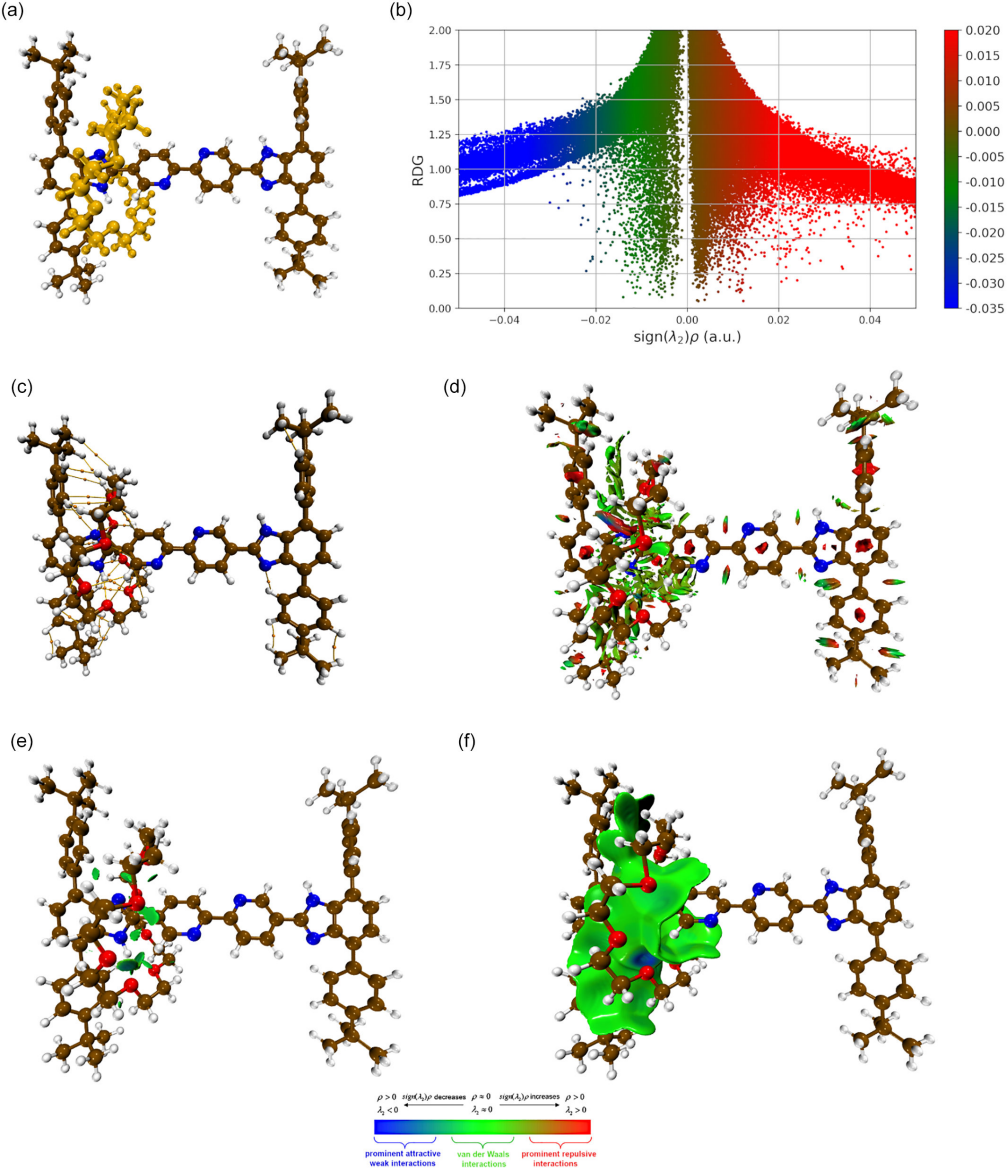
(a,b) The symmetric H‐shaped [2]rotaxane (X‐ray structure, CCDC 2 248 267) [47] and its RDG versus sign(*λ*_2_)*ρ* bidimensional (NCI) plot; (c,d) QTAIM bond paths marked with orange ropes and 3D NCI isosurfaces. [From Ref. [47], (a–d)]. IGMH δ*g* ^inter^ plots with two different isovalues: 0.01 a.u. (e) and 0.001 a.u. (f). Carbon atoms are reported in ochre, nitrogen atoms in blue, oxygen atoms in red, and hydrogen atoms in white.

The first case study illustrates how topological analyses can be employed not only to identify the noncovalent interactions stabilizing mechanically interlocked architectures but also to extract design principles governing macrocycle recognition and controlled shuttling [47]. Using an H‐shaped [2]rotaxane as a representative example (Scheme 1a) [48], the combined QTAIM/IGMH analysis reveals how different classes of supramolecular interactions cooperate to regulate both the equilibrium position of the macrocycle and its dynamic motion. Taking the published X‐ray structure as the reference, the QTAIM analysis—at the B3LYP(D3)/cc‐pVTZ level [33, 51, 52, 53]—identified an extensive network of bond critical points associated with hydrogen bonding and dispersive interactions, providing a quantitative picture of the supramolecular “interaction network” responsible for macrocycle stabilization. The strongest host–guest contacts are two (ether)O···H─N hydrogen bonds (distances 2.06 and 2.22 Å; *ρ*(***r***_***cp***_) ≈ 0.014–0.020 *e·a*_0_^−3^) that anchor the wheel at the benzimidazolium (Bzi) station, while a diverse web of weaker C─H···O and O···C(sp^2^) van der Waals contacts stabilizes the passing of the ring over the Bipy unit. In this respect, in Figure 1a–d, let me report the noncovalent results previously published with the addition of IGMH plots with two different δ*g* ^inter^ isosurfaces, depicted in panel 1e,f. This further analysis, while supporting the experimental [48] and theoretical [47] investigations, one more time clearly remarks the most prominent attractive noncovalent interactions regulating the preferential position of the **24C8** ring over the symmetric Bzi‐Bipy‐Bzi molecular thread. As a matter of fact, when a value of δ*g* ^inter^ = 0.01 a.u. is imposed, the two strongest [host]O···H─N[guest] hydrogen bonds are properly represented in Figure 1e. This analysis is carried out considering the thread and the macrocycle as separate fragments. A reduction of an order of magnitude in the δ*g* ^inter^ descriptor (from 0.01–0.001 a.u.) even reveals the contextual presence of weaker wdW forces further stabilizing the macrocycle over the associated Bzi station (see Figure 1f). The smoother appearance of the IGMH‐derived isosurfaces compared with those obtained using the NCI method can be attributed to the intrinsic regularization introduced by the IGMH approach, which therefore provides less jagged and more physically meaningful isosurfaces [45, 46, 54].

Another key result of this computational study is the quantum‐mechanical characterization of the Zn(II)‐induced shuttling mechanism. A previous DFT work had proposed an octahedral intermediate in which the metal coordinates two Bipy nitrogen atoms, one equatorial oxygen of **24C8**, one equatorial and two axial crown‐ether oxygens, and one DMF carbonyl [48]. By reoptimizing this species at the higher C‐PCM(DMF)/B3LYP(D3)/cc‐pVTZ level and examining *H*(***r***) contour maps (Figure 4 of Ref. [47]), topological descriptors clearly distinguish the stronger covalent Zn–bipyridyl interactions from the weaker and more labile Zn–macrocycle contacts, explaining why the latter remain sufficiently reversible to sustain shuttling: the Bipy─Zn bonds [*H*(***r***_**cp**_) ≈ −0.012 hartree·*a*_0_^−3^) and the Zn─O(DMF) bond [*H*(***r***_**cp**_) ≈ −0.005 hartree·*a*_0_^−3^] are covalent in character, whereas the Zn─O(**24C8**) contacts [*H*(***r***_**cp**_) ≈ −0.001 hartree·*a*_0_^−3^] are only marginally negative confirming that the crown ether forms weak, reversible bonds with the metal ion that allow continued shuttling, consistent with the observed 2.2 s^−1^ rate. The accompanying electrostatic surface potential (ESP) reveals how *cis*–*trans* isomerization of the Bipy unit gates the local electrophilicity at the nitrogen atoms (from −61.3 to −21.5 kcal/mol), rationalizing the selective Zn^2+^ and Pt^2+^ complexation chemistry observed in the laboratory [48]. From a broader perspective, this study illustrates how computational topology can move beyond the simple characterization of intermolecular contacts. By identifying which interactions are persistent, reversible, or cooperative, these analyses establish practical design principles for molecular machines. In particular, they emphasize that efficient shuttling requires a delicate balance between strong recognition motifs that ensure selective binding and weaker secondary interactions that preserve structural flexibility.

## 3 All‐Atoms MD and DFT Characterization of the Same Rotaxane: Dynamics and Pt(II) Locking

While the previous investigation established the static interaction network responsible for macrocycle recognition, this second study demonstrates how the integration of MD and quantum‐chemical topology can elucidate the dynamic behavior of molecular machines [49, 55]. More importantly, it illustrates how combining complementary computational approaches enables the transition from structural characterization to the mechanistic understanding of controlled molecular motion. The time‐resolved N─H···O(ether) distances (Figure 2 of Ref. 49) confirm that a cooperative triad of hydrogen bonds anchors the **24C8** ring at the Bzi station throughout the classical sampling, with the three participating oxygen atoms exhibiting complementary occupancies consistent with the experimentally acquired ^1^H NMR spectra [48].

**FIGURE 2:**
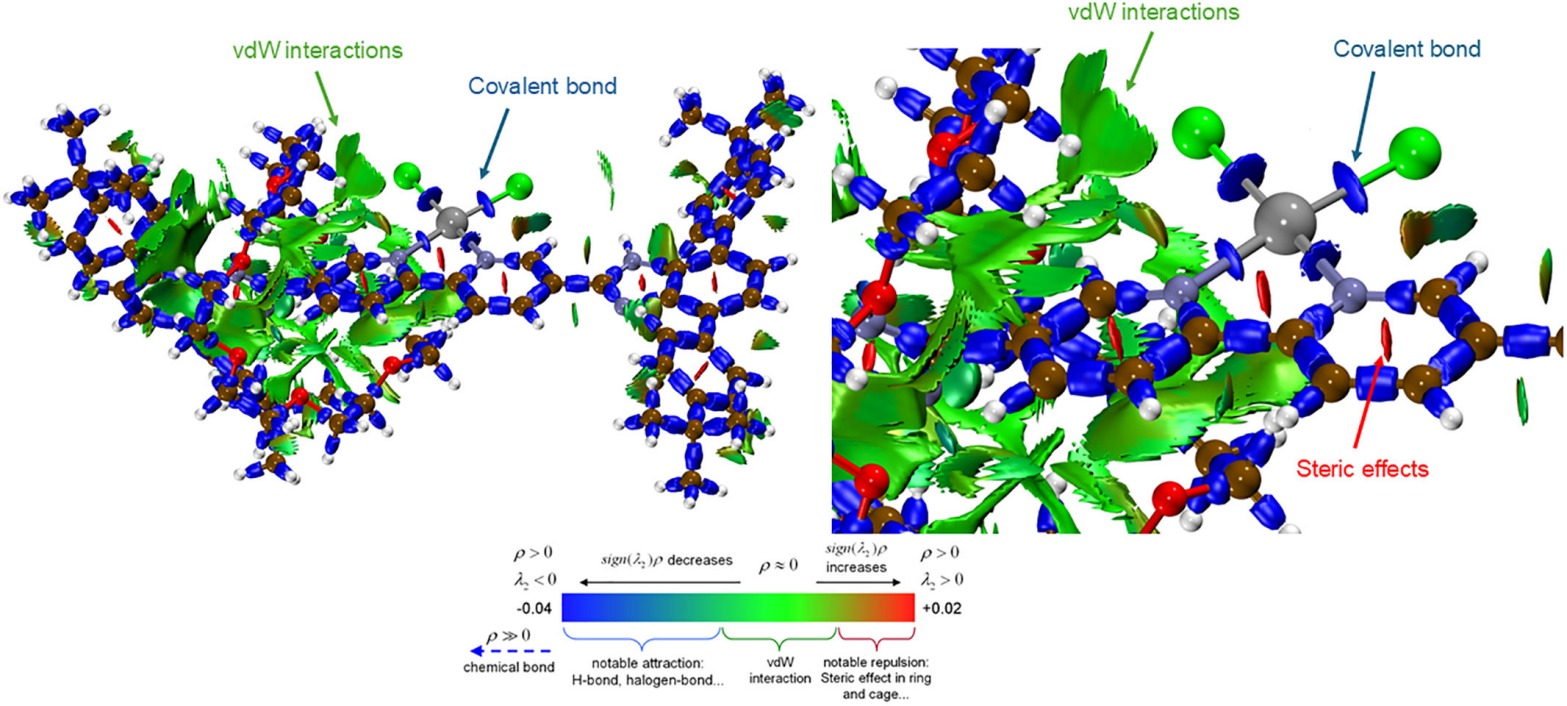
Isosurface maps of IRI = 1.0 for the Stop–[Bzi(**24C8**)–Bipy(PtCl_2_)–Bzi]–Stop supramolecular [2]rotaxane at C–PCM(DMF)/B3LYP(D3)/cc–pVTZ level of computation. sign(*λ*_2_)*ρ* is mapped on the isosurfaces according to coloring method used; a grid spacing of 0.12 *a*_0_ is applied. Carbon atoms are reported in ochre, nitrogen atoms in light blue, oxygen atoms in red, hydrogen atoms in white, chlorine atoms in green, and the platinum atom in silver.

Umbrella sampling (16 windows, 20 ns each, harmonic restraint 30 kJ mol^−1^ Å^−2^) provided the Potential of Mean Force (PMF) [39] for the Bzi→Bzi translocation of the **24C8** ring in CH_2_Cl_2_ dilute solution. The estimated free‐energy barrier, Δ*G* ^‡^ ≈ 11.2 ± 0.3 kcal/mol, compares favorably with the experimental activation free energy of 15.1 kcal/mol extracted from variable‐temperature ^1^H NMR experiments, and it improves substantially over an earlier DFT‐only estimate of ~24 kcal/mol in DMF [47]. Crucially, the profile reveals that the macrocycle remains predominantly localized over the Bzi station mainly through a cooperative hydrogen‐bonding network. Free‐energy calculations further revealed that shuttling proceeds through a metastable intermediate (8.6 ± 0.2 kcal/mol above the Bzi minimum) stabilized by multiple weak interactions, emphasizing the cooperative nature of molecular recognition in mechanically interlocked systems: a network of 16 BCPs connecting Bipy with the crown ether is appreciated, with two shortest C(sp^2^)─H···O contacts at the borderline between noncovalent and weak partial‐covalent character (see Table 1 of Ref. [48] for major details). This represents an important design principle for future molecular shuttles, where interaction cooperativity may be more relevant than maximizing the strength of individual recognition motifs.

A second conceptual contribution concerns the mechanism by which Pt(II) inhibits molecular shuttling [48]. Rather than merely forming coordination bonds with the Bipy unit, the metal center reorganizes the electronic structure of the entire recognition site, generating a square‐planar Stop‐[Bzi(**24C8**)–Bipy(PtCl_2_)–Bzi]–Stop complex that actually introduces steric and electronic constraints preventing macrocycle displacement (see Scheme 1b). QTAIM‐based indicators at the Pt^2+^–N(Bipy) BCPs [*ρ*(***r***_**cp**_) ≈ 0.12 *e·a*_0_^−3^; *H* (***r***_**cp**_) ≈ −0.042 hartree·*a*_0_^−3^; *δ*(*X*,*Y*) ≈  0.81] and at the Pt^2+^–Cl^‒^ BCPs [*ρ*(***r***_**cp**_) ≈ 0.09 *e·a*_0_^−3^; *H*(***r***_**cp**_) ≈ −0.024_5_ hartree·*a*_0_^−3^; δ(*X*,*Y*) ≈ 0.90] unambiguously assign these mutual interactions as covalent bonds. In this respect, the delocalization index, δ(*X*,*Y*), is also used to provide a direct measure of the number of electrons that are exchanged between *X* and *Y* atomic species, giving in essence the fractional number of electron pairs shared by two interacting atoms [49]. The *H*(***r***) contour maps over the σ[Cl–Pt–Cl] and σ[N–Pt–N] planes display uninterrupted corridors of negative values connecting the metal cation to its four ligands, thereby providing the first‐principles proof that the PtCl_2_ complex creates an insurmountable steric and electronic barrier to **24C8** shuttling. It is worth noting that the computed Pt–Cl stretching frequencies (312 and 326 cm^−1^ in the full [2]rotaxane) reproduce literature values [56, 57, 58] for analogous Pt(II) complexes and validate the overall theoretical approach implemented [48]. The presence of a covalent framework connecting the Pt^2+^Cl_2_ moiety to the Bipy complexation site is still reinforced by deriving the interaction region indicator (IRI),^3^ [59] which represents a real‐space descriptor capable of revealing both chemical bonds and weak noncovalent interactions into a single postprocessing analysis [60]. The isosurface maps of IRI = 1.0 reported in Figure 2, presented here for the first time, well reveal regions corresponding to each covalent bond, and noncovalent forces arising from steric repulsions and attractive vdW interactions can also be visualized. We can conclude from this inspection of the reported Stop–[Bzi(**24C8**)–Bipy(PtCl_2_)–Bzi]–Stop supramolecular adduct that the hypothesized covalent planar square (Bipy)–N_2_–Pt^2+^Cl_2_ complex, parallel to the molecular axle, should be effective in locking the shutting movement as observed via ^1^ H‐NMR measurements in DMF solvent [48]. Moreover, the newly introduced IRI analysis further demonstrates the value of combining complementary real‐space descriptors. Unlike QTAIM, which focuses on individual critical points, IRI simultaneously visualizes covalent bonds, steric effects, and weak intermolecular interactions within a unified framework. This integrated representation considerably facilitates the interpretation of complex mechanically interlocked architectures, suggesting that future computational investigations should increasingly exploit complementary visualization techniques rather than relying on a single descriptor.

## 4 DFT Investigation of a pH‐ and Redox‐Driven Tristable [2]Rotaxane

The last paper selected [50] is focused on a more complex supramolecular platform: a recently synthesized tristable [2]rotaxane in which a dibenzo‐24‐crown‐8 (**DB24C8**) macrocycle can be positioned at three distinct recognition sites—ammonium (AmH^+^), bipyridinium (Bpy^2+^), or triazolium (Trz^+^) species—through fully orthogonal pH and electrochemical stimuli (see also Scheme 1c [27]). This final case study extends the discussion from bistable to multistable molecular machines, illustrating how modern computational chemistry can predict the interplay between multiple external stimuli and the corresponding structural response. Three equilibrium states [AmH^+^(**DB24C8**)–Bpy^2+^–Trz^+^, Am–Bpy^2+^(**DB24C8**)–Trz^+^, Am–Bpy–Trz^+^(**DB24C8**)] are individually relaxed and TD‐DFT [35, 50] absorption spectra are computed and convoluted with Gaussian lineshapes [61, 62]. The selective reduction of Bpy^2+^ → Bpy is manifested as a new absorption band at ~375 nm attributable to an electronic excitation across the planarized bipyridine moiety, and the accompanying dihedral angle change from 47° (Bpy^2+^, twisted) to ~0° (Bpy, planar) is fully reproduced.

Electrostatic potential (ESP) surfaces [63] provided an intuitive rationalization of the sequential ring translocation: the AmH^+^ unit presents the highest positive ESP (up to 240 kcal/mol on its vdW surface), directing the macrocycle to the ammonium station in the first state; deprotonation attenuates this selectivity, shifting the ESP maximum to Bpy^2+^; further electrochemical reduction eliminates the bipyridinium charge, leaving Trz^+^ as the sole positive site. QTAIM + IGMH analysis of the three host–guest adducts (Table 1, S4, S5 and Figure 5 of Ref. [50]) reveals the full interaction repertoire at the nanoscale level: at the AmH^+^ station, two medium‐strong N─H···O(ether) hydrogen bonds [distances 1.68 and 1.76 Å; *H*(***r***_**cp**_) ≈ −0.004 and −0.002 hartree·*a*_0_^−3^; binding energies (B_Es_) [64] −17.0 and −15.0 kcal/mol] dominate alongside a rich network of weaker C─H···O and C···O contacts; at Bpy^2+^, the anchoring is achieved through an array of C(sp^2^)─H···O(ether) CH···O hydrogen bonds (B_E_ range −12.2 to −3.1 kcal/mol); at Trz^+^, direct C(sp^2^)···O(ether) electrostatic contacts between positively charged carbon atoms (~+0.45*e*) and negatively charged ether oxygens (~−0.97*e*) supplement weak aliphatic C─H···O hydrogen bonds. Among other things, this progressive diminution of H‐bond strength across AmH^+^ > Bpy^2+^ > Trz^+^ is fully consistent with the ^1^H NMR‐observed affinity order in CH_2_Cl_2_ at 298 K [27].

**FIGURE 3:**
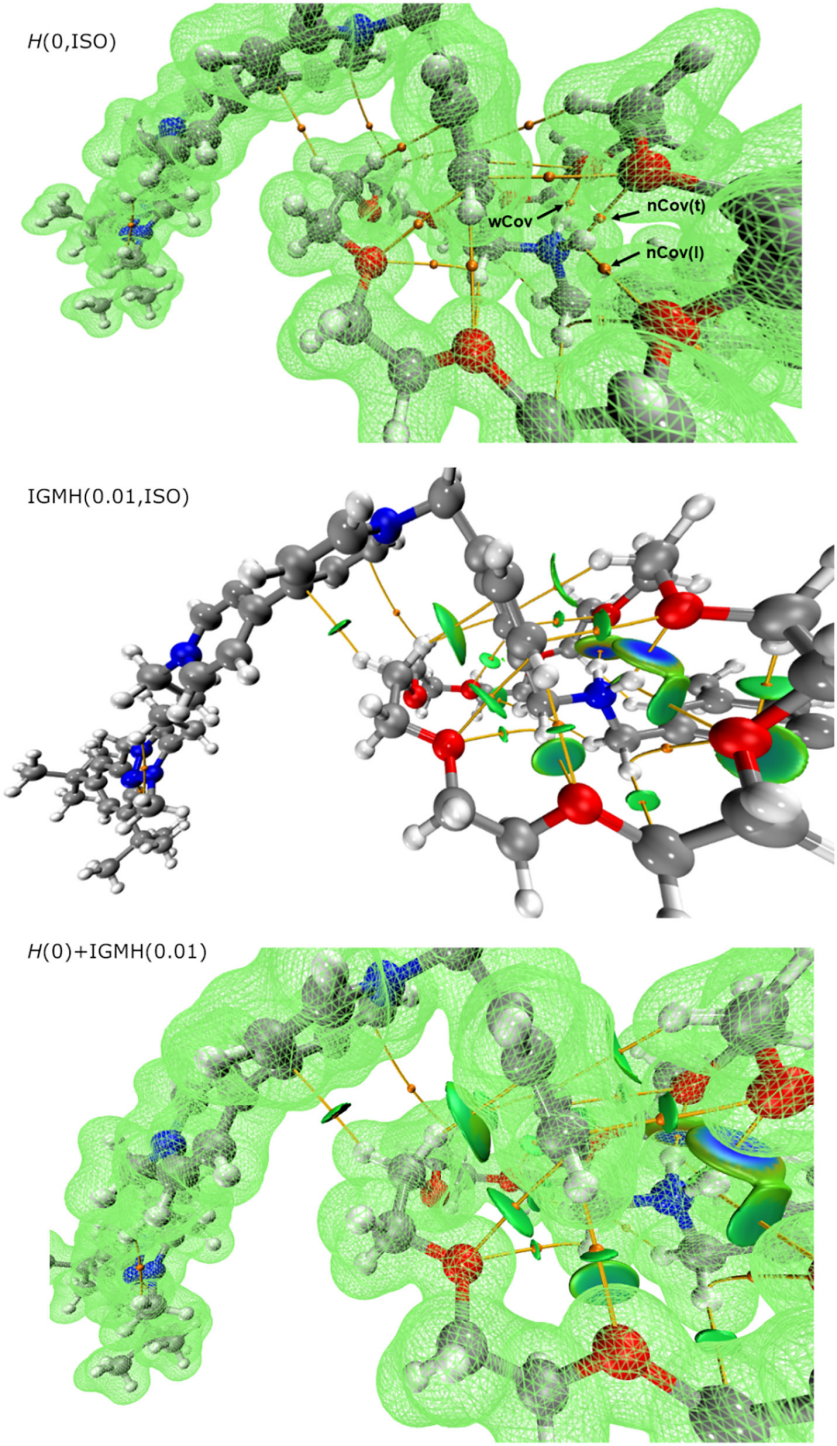
Upper panel: C‐PCM(CH_2_Cl_2_)/B3LYP(D3)/6‐311G** *H*(0,ISO) isosurface of the AmH^+^(**DB24C8**)–Bpy^2+^–Trz^+^ tristable [2]rotaxane. Middle panel: IGMH δ*g* ^inter^ = 0.01 a.u. isosurfaces always at the C‐PCM(CH_2_Cl_2_)/B3LYP(D3)/6‐311G** level of computation. Lower panel: *H*(0,ISO)+IGMH(0.01,ISO) isosurfaces. A grid spacing of 0.12 *a*_0_ is applied. Carbon atoms are reported in silver, nitrogen atoms in light blue, oxygen atoms in red, and hydrogen atoms in white.

In the present context, the predictive power of numerical simulations at DFT cost is even remarked in Figure 3 where we have reanalyzed the experimentally observed high selectivity enabling the formation of the AmH^+^(**DB24C8**)–Bpy^2+^–Trz^+^ supramolecular complex [27]. More specifically, in the same figure we graphically merge the results obtained combining the GLED model recently proposed in literature [65, 66], which relies on the graphical representation of the local electron energy density *H*(***r***), with the QTAIM + IGMH couple. The GLED method is seen to self‐consistently describe the chemical bond in terms of the three major categories typically used: the covalent bond, the ionic bond, and the noncovalent interactions driving the stability of more or less complex intermolecular aggregates. Interestingly, the major character of the bond (covalent or noncovalent) can be inferred by the visual inspection of the plotted *H*(***r***), particularly the 3D *H*(***r***) = 0 isosurface [*i.e.*, the *H*(0,ISO)]. When interacting moieties overlap their outermost *H* ^*+*^(***r***) regions, but their valence *H*(0,ISO) do not touch each other, and their enclosed valence *H* ^‐^(***r***) contour lines remain well separated and the underlying contact features a noncovalent (nCov) character. On the other hand, when the two fragments overlap so to produce an outer *H*(0,ISO) encompassing the nuclei of both fragments and enclosing the electrons with negative energies populating the valence *H* ^‐^(***r***) binding domain (VBD) of their precursor moieties, the underlying interactions may be characterized as weakly covalent (wCov). The plot of the *H*(0,ISO) displayed for the first time in Figure 3 (upper panel) let us plausibly suppose the presence of three different mutual interactions along the derived [AmH^+^]N─H···O(ether)[ **DB24C8**] QTAIM bond paths (BPs). As a matter of fact, it can be seen that a wCov contact may be appreciated for the shortest N─H·(1.68 Å)·O(ether) contact; in contrast, the longest contact (2.28 Å) presents a (+3,−1) bond critical point (BCP, ***r***_**cp**_) located outside the *H*(0,ISO) isosurface, which is indicative of a noncovalent interaction. More specifically, on the basis of the associated *ρ*(***r***_**cp**_) values (0.0478 and 0.0131 *e*·*a*_0_^−3^), these two contacts feature dissociation energies (*D*_e_) [66] of 17.0_2_ and 5.3_9_ kcal/mol, respectively. Consequently, the interaction [AmH^+^]N─H···O(ether)[ **DB24C8**] pattern at 2.28 Å is reported as nCov(l = loose) in Figure 3 (upper panel). Moreover, it is interesting to highlight that the contact with N–H···O distances at 1.76 Å lies on the boundary between a wCov and a nCov(t = tight) interaction, since the interacting fractions overlap their outermost *H* ^+^(***r***) regions, but their valence *H*(0,ISO) barely touch each other leaving the enclosed valence *H* ^*−*^(***r***) regions slightly separated. However, this contact is labeled nCov(t) in Figure 3 and has an estimated *D*_e_ of 15.0_1_ kcal/mol. The IGMH plot reported in the middle panel of the same figure with a δ*g* ^inter^ of 0.01 a.u. results in line with the picture derived with the GLED model. Please note that the coloring scheme adopted is the same as that already applied in Figure 1. At last, for the sake of completeness, the lower panel in Figure 3 reports a combined *H*(0,ISO)+IGMH(0.01,ISO) plot, which thus allows the nature and intensities of the supramolecular interactions under investigation to be assessed on a point‐by‐point basis.

## 5 Cross‐Cutting Themes and Methodological Advances

Reading across these recent studies, several unifying themes emerge. First, the QTAIM/*H*(***r***) framework proves to be a highly discriminating diagnostic: the sign and magnitude of the *H*(***r***) at bond critical points reliably distinguish covalent metal–ligand or hydrogen bonds [*H*(***r***_**cp**_) ≪ 0] from medium‐strength hydrogen bonds [*H*(***r***_***cp***_) ≈ 0 or slightly negative] from purely electrostatic van der Waals contacts [*H*(***r***_**cp**_) > 0], enabling a quantitative classification that goes beyond distance‐only criteria between functional groups. Second, the IGMH visualization of δ*g* ^inter^ isosurfaces, colored by sign(*λ_2_*)*ρ*, provides a physically transparent 3D map of the noncovalent interaction topology in systems containing hundreds of atoms. Third, the combination of MD free‐energy calculations (umbrella sampling/WHAM) with static DFT + QTAIM snapshots bridges the timescale gap between quantum‐mechanical accuracy and thermodynamic completeness, yielding barriers in quantitative agreement with NMR kinetics. Fourth, the explicit treatment of solvent polarity via C‐PCM and of dispersive interactions via DFT‐D3 is consistently shown to be essential for reproducing the correct energetic ordering of translational isomers in low‐polarity solvents such as CH_2_Cl_2_ and DMF. Positioning this contribution within the broader computational literature, theoretical and computational investigations of rotaxane‐ and catenane‐based ion recognition, host–guest chemistry, and binding selectivity constitute a vigorously active research area extending well beyond the systems discussed above [67, 68, 69, 70]. Furthermore, beyond QTAIM‐ and IGM‐based real‐space descriptors, modern energy‐decomposition approaches (EDA) are increasingly employed to quantify binding selectivity in MIMs in energetic rather than purely topological terms [71, 72, 73, 74]. Machine‐learning‐accelerated exploration of MIMs energy landscapes represents an emerging complementary direction [75, 76, 77, 78]; a recent nudged‐elastic‐band study coupled to a machine‐learned potential traced the threading (interlocking) pathway of a rotaxane precursor, providing atomistic access to the mechanical‐bond‐formation step that static DFT alone cannot resolve [79]. Another interesting and emerging field of applications regards the rotaxane‐assisted ion translocation across lipid membranes utilizes MIMs where a macrocyclic “ring” shuttles along a transmembrane “axle”. Acting like a microscopic cable car or a shuttle‐relay mechanism, the ring binds ions at specific recognition sites and transports them across hydrophobic lipid bilayers [80, 81, 82].

Without any doubt, as synthetic rotaxane architectures grow in complexity—from bistable two‐station shuttles to tristable three‐station devices and from single‐stimulus to multiorthogonal switching—the demands placed on computational and theoretical modeling are correspondingly intensified. Static DFT calculations of isolated host–guest pairs, while still informative, are actually no longer sufficient to capture the thermodynamic and the conformational shaping adopted by molecular machines and devices operating in solution at finite temperature conditions. Beyond the immediate rotaxane context, the computational strategies herein highlighted are directly exportable to the broader landscape of emerging nanoscale devices. Molecular pumps that use chemical fuels to maintain nonequilibrium distributions of macrocycle positions, catenane‐based molecular gyroscopes, and rotaxane‐threaded metal–organic frameworks for heterogeneous catalysis all pose, at their core, the same fundamental question addressed here: *What is the hierarchical interplay of noncovalent forces and nanosolvation landscapes through which molecular machines translate single‐molecule dynamics into a macroscopic function?* Answering this fundamental question with atom‐level resolution is the essential first step toward a genuinely predictive and ecosustainable, computation‐guided investigation of next‐generation molecular machines and devices of increasing complexity and functionalities. In conclusion, this contribution highlights some significant advancements in the theoretical characterization of artificial molecular machines of a certain complexity, bridging electronic‐structure theory, MD, and state‐of‐the‐art supramolecular computational chemistry algorithms. The presented methodologies therefore constitute a valid framework for understanding and designing functional interlocked architectures with tailored properties.

## Disclaimer

The opinions expressed in this publication are the view of the author(s) and do not necessarily reflect the opinions or views of ChemistryOpen, the Publisher, ChemistryEurope, or the affiliated editors.

## Conflicts of Interest

The author declares no conflicts of interest.

## Data Availability

The data that support the findings of this study are available from the corresponding author upon reasonable request.

## Endnotes

1 The *ρ*(***r***) is defined by the following equation:

(1)

where *η* *_i_* is the occupation number of the natural Kohn–Sham (KS) orbital *φ* *_i_*, in turn expanded as a linear combination of the atomic basis functions [32]. The *H*(***r***) is the sum of the kinetic energy density *G*(***r***) and the potential energy density *V*(***r***) [29]:

(2)
H(r)=G(r)+V(r)

The presently employed definition [32, 41, 42, 43] of the *G*(***r***) is given by the equation:

(3)

with the sum running over all the occupied natural orbitals *φ* *_i_* of occupation numbers *η* *_i_*. The potential energy density *V*(***r***) is evaluated from the local form of the virial theorem:

(4)
$$V(r)=\frac{1}{4}\nabla^{2}\rho (r)‐2G(r)$$

The values of the *G*(***r***) and *V*(***r***) are diagnostic for evaluating if interactions are controlled by a local reduction of the potential energy or by a local excess in the kinetic energy [*G*(***r***) > 0 and *V*(***r***) < 0, see Equations (3) and (4)]. The total energy density *H*(***r***), see Equation (2), which is negative for interactions featuring a significant sharing of electrons, enables the detection of the covalent or partially covalent character along interaction patterns. At last, the Laplacian of the density [∇^2^ *ρ*(***r***)]—as derived from the trace of the Hessian matrix of the *ρ*(***r***)—allows an analysis of the curvatures of the *ρ*(***r***) at any estimated BCPs along the principal axes. In addition, we would like to specify that the independent gradient model (IGM) and independent gradient model based on Hirshfeld partition (IGMH) are closely related methods for visualizing and quantifying noncovalent interactions, but they differ fundamentally in how they define the underlying electron density. IGMH replaces the promolecular density with the actual electron density obtained from a quantum mechanical calculation. The Hirshfeld partitioning scheme applied defines a weighted atomic density:

(5)
$$\rho_{i}^{\mathrm{Hirsh}}(r)=\rho (r)\cdot w_{i}(r)$$

where *ρ*(***r***) is the actual electron density while the Hirshfeld weighting function of the atom *i* is defined as:

(6)
$$w_{i}(r)=\frac{\rho_{i}^{0}(r)}{\rho^{0}(r)}=\frac{\rho_{i}^{0}(r)}{\sum_{j}^{\mathrm{N atoms}}\rho_{j}^{0}(r)}$$

where index *j* loops over all atoms, the *ρ* ^0^(***r***) in the denominator is a promolecular density, and $\rho_{i}^{0}$ stands for spherically averaged density around atom *i* in its unperturbed (free) state [45, 46]. In the IGM model, the *ρ* ^0^(***r***) is defined on densities of atoms in their free states under the so‐called promolecular approximation. On the contrary, in the IGMH method, the free‐state atomic density (*e.g.*, $\rho_{i}^{0}$) in the IGM description is replaced with $\rho_{i}^{\mathrm{Hirsh}}$. The key descriptor of IGM‐based models is the *δ* *g* ^inter^ function, which isolates intermolecular (or interfragment) interactions:

(7)
$$\delta g^{\mathrm{inter}}=\left|\nabla \rho^{\mathrm{IGM}}\left(r\right)\right|‐\left|\nabla \rho \left(r\right)\right|$$

where *ρ* ^IGM^(***r***) is the electron density constructed as the sum of the densities of isolated promolecular fragments:

(8)

In the Hirshfeld‐based version, the function is modified to account—starting from a converged electronic wavefunction—for atomic partitioning as follows:

(9)

The principle is brilliant in its simplicity; if there were no interaction between *i* (fragment 1) and *j* (fragment 2) couple of atoms, the additive property of gradients would apply

(10)
$$g_{i\text{,}j}(r)\;=\left|\nabla (\rho_{i}(r)+\rho_{i}(r)\right)|=\left|\nabla \rho_{i}(r\right)|+\left|\nabla \rho_{j}(r)\right|=g_{i\text{,}j}^{\mathrm{IGM}}$$

and therefore $\delta g_{\mathrm{inter}}^{\mathrm{Hirsh}}$ = 0; when interaction occurs, the electron density is redistributed and

(11)
$$\left|\nabla (\rho_{i}(r)+\rho_{j}(r))\right|\;\neq \;\left|\nabla \rho_{i}(r\right)|+\left|\nabla \rho_{j}(r)\right|$$

The difference measures how much the combined system deviates from the behavior of two isolated, noninteracting atoms. In the IGMH analysis, the *ρ* *_i_* terms over isolated fragments in the above expressions correspond to $\rho_{i}^{\mathrm{Hirsh}}$. The $\delta g_{\mathrm{inter}}^{\mathrm{Hirsh}}$ related isosurfaces are then used to visually appreciate the interaction regions, while the function sign(*λ*_2_)*ρ*(***r***) is superimposed on the estimated isosurfaces to visually characterize the nature of the chemical interactions. The latter indicates the product of the electron density, *ρ*, and the sign of the second largest eigenvalue of the Hessian matrix of the electronic density; *ρ* is generally positively correlated with the interaction force, while sign(*λ*_2_) has instead the ability to distinguish whether the interactions are attractive or repulsive. Thus, interaction patterns are visualized in a 3D space by plotting a chosen low‐value isosurface of the $\delta g_{\mathrm{inter}}^{\mathrm{Hirsh}}$, colored by the sign(*λ*_2_)*ρ*(***r***).

2 In the QTAIM, the delocalization index *δ*(*X*,*Y*) quantifies the number of electron pairs shared (delocalized) between two atomic basins Ω*_x_* and Ω*_y_* and is therefore used as a quantum‐mechanical measure of bond order or noncovalent electron sharing. It is formally derived from the exchange–correlation density and is defined as

(12)
$$\delta (X,Y)=-2\int \left\{\Omega_{x}\right\}\int \left\{\Omega_{y}\right\}\Gamma_{xc}(r_{1},r_{2})dr_{1}dr_{2}$$

where (i) Ω*_x_* and Ω*_y_* are the atomic basins of atoms *X* and *Y*, defined by the zero‐flux surfaces in the gradient of the electron density; (ii) Γ*_xc_* (*r*_1_,*r*_2_) is the exchange–correlation density, obtained from the difference between the total pair density and the classical (Coulombic) product *ρ*(***r***_1_)*·ρ*(***r***_2_); and (iii) the double integral is evaluated by integrating *r*_1_ over Ω*_x_* and *r*_2_ over Ω*_y_*. Larger *δ*(*X*,*Y*) values indicate greater electron sharing (and thus stronger covalent character) between *X* and *Y* atoms, while smaller values are typical of closed‐shell or noncovalent interactions, making *δ*(*X*,*Y*) a natural complement to descriptors such as IGMH and GLED when characterizing bonding versus noncovalent regions at a nanoscale level.

3 The IRI, introduced by Lu and Chen, is a real‐space density‐based descriptor designed to simultaneously reveal both chemical bonds and weak intermolecular interactions within a unified framework. Mathematically, IRI is defined as the ratio between the norm of the electron‐density gradient and the electron density raised to an empirical exponent (typically 1.1), thereby reducing the excessive divergence exhibited by the reduced density gradient (RDG) in high‐density regions:

(13)
$$\mathrm{IRI}(r)=\frac{\left|\nabla \rho (r)\right|}{{\left|\rho (r)\right|}^{a}}$$

As a result, IRI provides continuous and chemically intuitive isosurfaces capable of identifying covalent bonds, hydrogen bonds, van der Waals interactions, steric repulsion, and other noncovalent interactions. Similar to NCI analysis, IRI isosurfaces are commonly colored according to the sign(*λ*_2_)*ρ* function. Owing to its simplicity, computational efficiency, and ability to simultaneously characterize bonded and nonbonded interactions, IRI has become an effective visualization tool in computational chemistry, materials science, and molecular recognition studies.
